# Longer Rodent Bioassay Fails to Address 2-Year Bioassay’s Flaws

**DOI:** 10.1289/ehp.11964

**Published:** 2008-12

**Authors:** Joseph Manuppello, Catherine Willett

**Affiliations:** People for the Ethical Treatment of Animals, Regulatory Testing Division, Norfolk, Virginia, E-mail: josephm@peta.org

In their commentary, Huff et al. (2008) proposed that exposing experimental animals to test substances *in utero* and for 30 months or until their natural deaths increases the sensitivity of bioassays, avoids false negative results, and strengthens the value and validity of results. Instead, longer exposure results in increased numbers of spontaneously arising tumors, as well as increased cost and animal suffering, while failing to address the bioassay’s fundamental flaws.

Although it is troubling when the bioassay produces false negative results, a far more pervasive problem is that of false positives. In our analysis of > 500 National Toxicology Program (NTP) bioassays [People for the Ethical Treatment of Animals (PETA) 2006], we found that more than half of the substances evaluated (259) produced evidence of carcinogenicity in at least one group of animals, but only about one-third of these (89) were subsequently classified as known or probable human carcinogens by the NTP itself. Even fewer of the substances, 40 and 16, respectively, were classified as carcinogens by the U.S. Environmental Protection Agency (EPA) and the International Agency for Research on Cancer (PETA 2006). This high false-positive rate is thought to be largely an indirect effect of increased cell proliferation in response to cell injury and death caused by the near toxic doses of test substances used in the bioassay (Gaylor 2005). Species-specific modes of action operating in rats or mice but not in humans, such as those mediated by 2μ-globulin, peroxisomes, and thyroid-stimulating hormone, also contribute to the high rate of false positives (Cohen 2004).

Huff et al. (2008) asserted that one of the “well-accepted observations” upon which the “relevance of experimental bioassays to humans” rests is that “findings from independently conducted bioassays on the same chemicals are consistent.” In fact, in a comparison of 121 bioassays from the NTP database with those in the published scientific literature, Gottmann et al. (2001) found that the studies produced consistent results only 57% of the time.

Huff et al. (2008) cited questions about the safety of aspartame raised by 3-year bioassays conducted by the Ramazzini Foundation to support their conclusions. Although they noted that the European Food Safety Authority (FSA) and the U.S. Food and Drug Administration (FDA) dispute these studies’ conclusions, the FSA’s Committee on Carcinogenicity (COC 2006) observed that

In view of the inadequacies in design of the [Ramazzini Foundation] study and the use of rats with a high concurrent infection rate, the COC considered that no valid conclusions could be derived from it.

Further, the COC noted that groups of animals fed aspartame had lower body weights and thus lived longer, which may have compromised the results by leading to an apparent increase in spontaneously arising tumors. Considering that lower body weights are typically observed among animals in the bioassay’s experimental groups, this is likely to generally confound the interpretation of longer bioassays.

We must stress that animals suffer during the bioassay: They live in the barren, stressful conditions of the laboratory—often including daily forced feeding or inhalation—and many also suffer from exposure to near toxic doses of test substances. These exposures often produce lethargy, anemia, diarrhea, weight loss, and other symptoms of sickness and distress. The proposal of Huff et al. (2008) to extend the length of the bioassay would obviously result in a proportional increase in this suffering.

Further, extending the bioassay runs counter to current trends in regulatory testing. Concern for the suffering of animals has caused regulatory agencies to review the usefulness of long-term studies, resulting in elimination of the 1-year dog toxicity test (U.S. EPA 2007) and an international effort to replace the two-generation reproductive toxicity test (Cooper et al. 2006). Huff et al.’s proposal thus clearly represents a step backward for toxicological science.

According to the NTP’s own estimates, each bioassay requires 5 years to plan, conduct, and evaluate; 860 animals to be killed; and $2–$4 million. As a result, the NTP has conducted an average of only 12 bioassays/year over the past several decades. Considering that humans are thought to be exposed to approximately 80,000 environmental toxicants (Ward et al. 2003), it would take more than 32 millenia, 68 million animals, and $160 billion to test them all at this rate. Once again, extending the length of the bioassay would only increase these already ridiculous numbers.

The time has clearly come for antiquated animal tests such as the bioassay to be abandoned in favor of modern, human-relevant methods such as epidemiologic studies, high-throughput *in vitro* methods, and computational toxicology.
